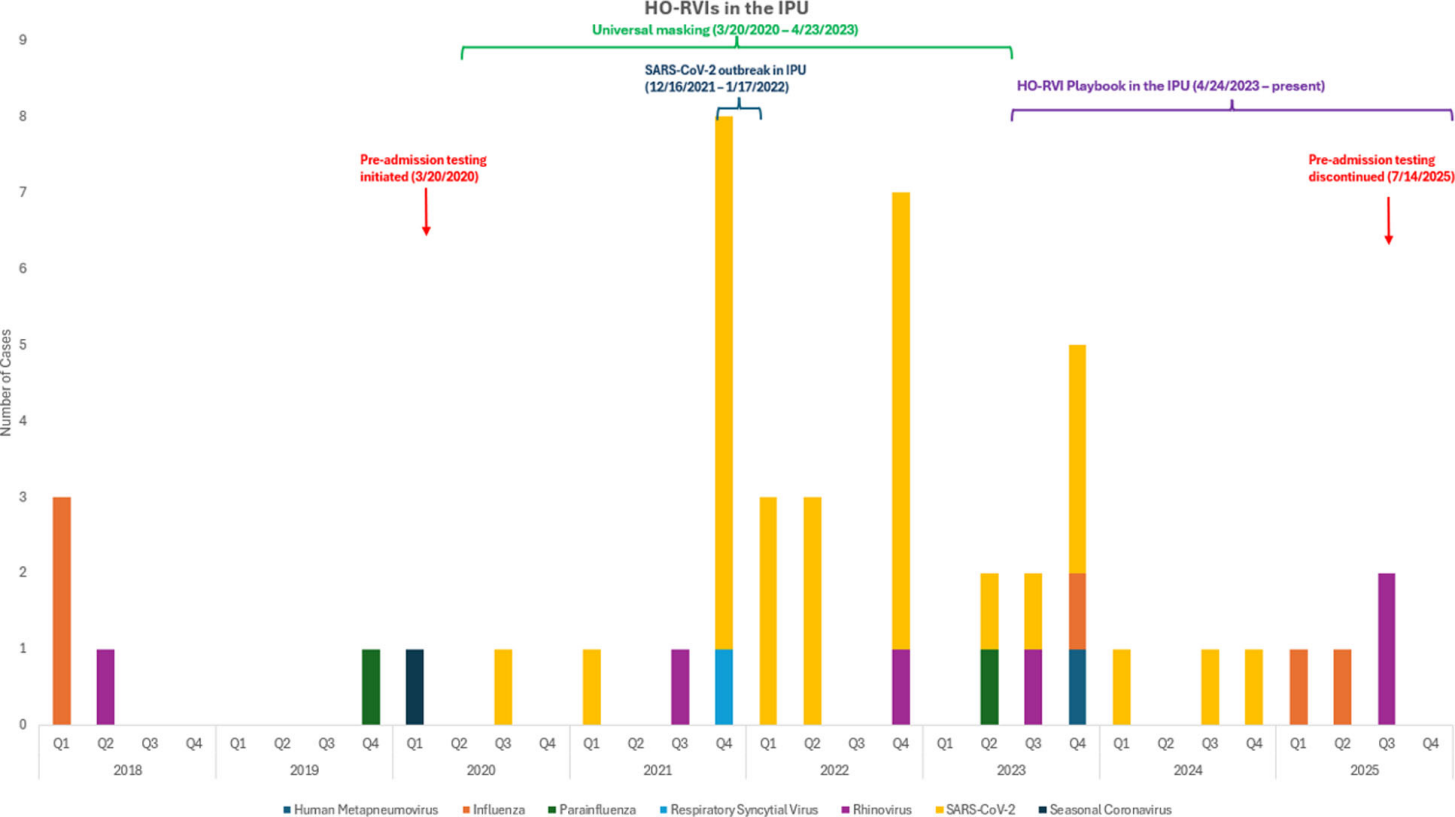# 46 Unpacking OPAT Risk: Factors Associated with Adverse Clinical Outcomes in an Academic OPAT Program

**DOI:** 10.1017/ash.2026.10484

**Published:** 2026-06-23

**Authors:** Alina Stancovici, Shardul Rathod, Grace Barajas, Maureen Bolon

**Affiliations:** 1 Northwestern Memorial Hospital

## Abstract

**Background:** Inpatient psychiatric units (IPUs) present unique challenges for prevention of respiratory virus illness (RVI) transmissions due to shared living spaces, group-based therapeutic activities, and limited ability to isolate or mask patients. Evidence-based infection prevention (IP) strategies in these settings are limited. We evaluated trends in hospital-onset (HO) RVIs on an IPU across pre-pandemic, pandemic, and post-pandemic masking eras and describe implementation of a unit-specific RVI mitigation playbook. **Methods:** We conducted a retrospective review of respiratory viral testing on the IPU from January 2018 through December 2025 at a 943-bed academic medical center. The IPU is comprised of 25 private rooms, two semi-private rooms, and shared common areas. Respiratory viral testing was performed on a reverse-transcriptase polymerase chain reaction (RT-PCR) assay (BioFire Respiratory Pathogen Panel, BioFire, Salt Lake City, UT or Xpert® Xpress CoV-2/Flu/RSV plus, Sunnyvale, CA) utilizing a nasopharyngeal swab. Patients were tested for respiratory viruses prior to IPU admission beginning March 2020 regardless of symptoms. Patients with positive results were not admitted to IPU due to the inability to accommodate isolation. Following a SARS-CoV-2 outbreak on the unit in December 2021-January 2022, a psychiatry-specific HO-RVI mitigation playbook was implemented to define response thresholds (Figure 1). HO-RVI was defined as symptom onset and/or a positive test occurring more than 48 hours after admission, with classification informed by virus-specific incubation periods based on published literature. A cluster was defined as two epidemiologically linked positive tests, including patient–patient or patient–healthcare worker combinations. An outbreak was defined as three or more patients and/or healthcare workers with HO-RVIs whose epidemiology indicates shared time and space. **Results:** On the IPU, HO-RVIs occurred sporadically (Figure 2). Since January 2022, no RVI outbreaks have occurred on this unit, despite periods without universal masking, continued identification of HO cases, and mitigation of seven RVI clusters. SARS-CoV-2 accounted for the majority of identified cases, though most were asymptomatic and detected through routine pre-procedural electroconvulsive therapy testing, without progression to outbreaks. **Conclusions:** Sustained prevention of RVI outbreaks on an IPU is achievable despite ongoing viral circulation and limited feasibility of patient isolation or masking. A tailored mitigation playbook incorporating early identification of cases and thresholds for implementing IP interventions may reduce outbreak risk while preserving the therapeutic milieu. These findings support development of setting-specific IP strategies for inpatient psychiatric environments. Additional studies are needed to inform best practices for preventing RVI transmissions in these settings.

**Figure:**
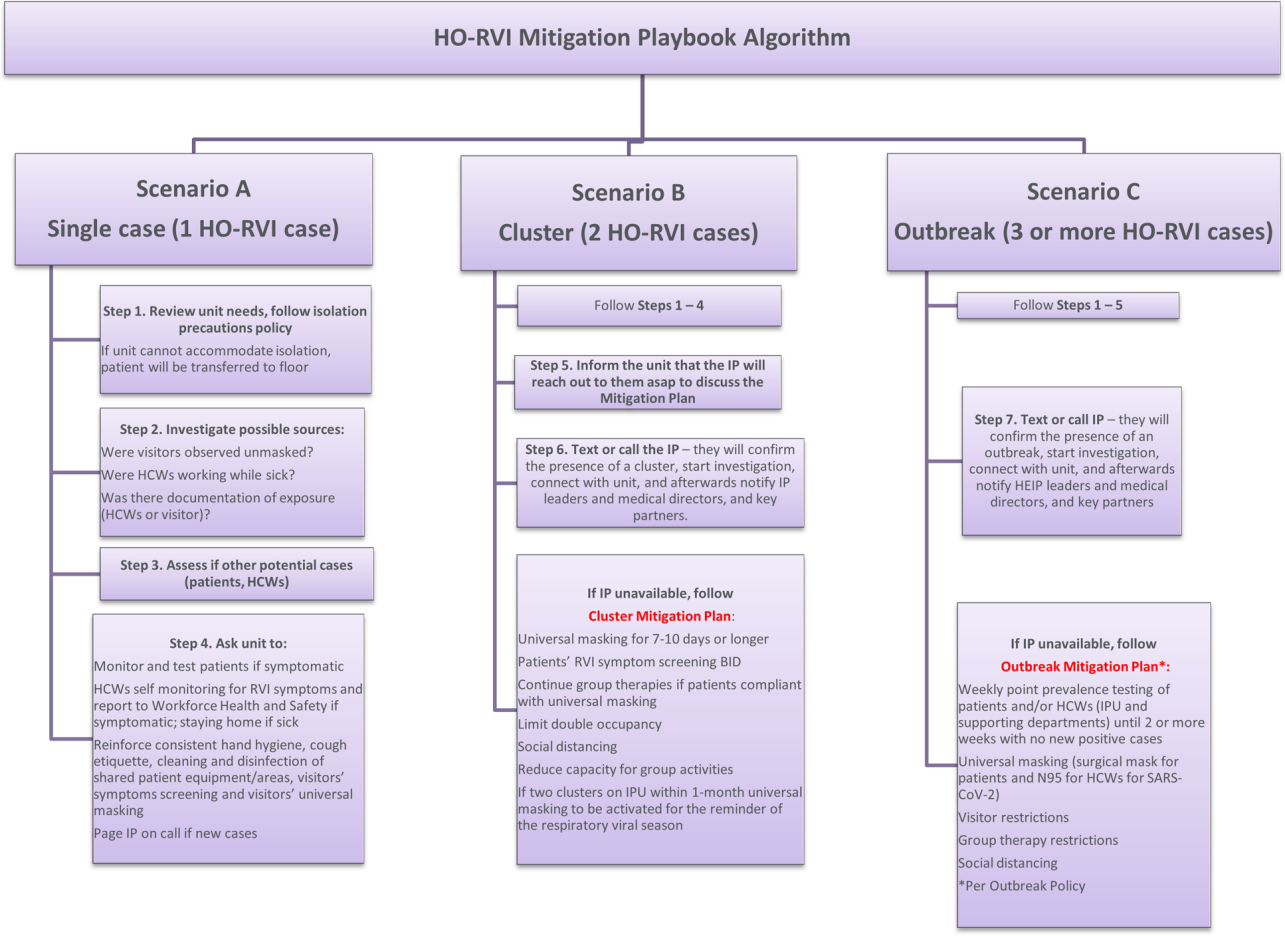

**Figure:**